# Pectoralis Major Pyomyositis in a Patient With Diabetes: A Case Report and Literature Review

**DOI:** 10.7759/cureus.53689

**Published:** 2024-02-06

**Authors:** Aya Tsunoda, Hideharu Nakamura, Takaya Makiguchi, Nana Tomaru, Satoshi Yokoo

**Affiliations:** 1 Department of Plastic and Reconstructive Surgery, National Hospital Organization (NHO) Takasaki General Medical Center, Takasaki, JPN; 2 Department of Oral and Maxillofacial Surgery, and Plastic Surgery, Gunma University Graduate School of Medicine, Maebashi, JPN

**Keywords:** diabetes, temperate region, morganella morganii, pectoralis pyomyositis, pyogenic myositis

## Abstract

Pyogenic myositis is a bacterial infection of skeletal muscle that is usually caused by Staphylococcus aureus and is common in tropical regions. Recently, this infection has also been reported in immunocompromised patients in temperate regions. The lower extremities and trunk are most affected, while involvement of the chest wall is rare. We report a case of pectoralis major pyomyositis caused by Morganella morganii in an 82-year-old Japanese man with type 2 diabetes mellitus who had undergone stenting for myocardial infarction. Four months prior to visiting our hospital, the patient became aware of pain in the right chest area, which gradually became swollen. One month before the visit, the pain and swelling had become more severe. At the visit, there was swelling in the right anterior thoracic region with a diameter of 10 cm and pain in the same region. On physical examination, his blood pressure was 133/64 mmHg, heart rate was 83 beats/min, and body temperature was 36.9℃. Initially, a sarcoma or other neoplastic lesion was suspected and a needle biopsy was performed. Pus was drained from the puncture site to collect wound culture. Needle biopsy of the lesion did not reveal any fungi or acid-fast bacteria, and a T-SPOT.TB test was negative. Computed tomography and magnetic resonance imaging suggested abscess formation under the pectoralis major muscle. A wound culture test detected Morganella morganii, and pectoralis major pyomyositis was diagnosed. Debridement was performed under general anesthesia. The necrotic pectoralis major muscle was excised, the abscess cavity was opened, and wound irrigation was performed. The postoperative course was good and the patient was discharged on the 16th postoperative day. There has been no recurrence in eight months postoperatively. Pectoralis major pyomyositis may not be relieved by antibiotics alone and may extend to deeper organs to form intrapleural abscesses. Therefore, prompt drainage should be performed to prevent serious complications in a case in which abscess formation is observed.

## Introduction

Pyogenic myositis is a bacterial infection of skeletal muscle from hematogenous spread. It can lead to abscess formation, sepsis, and other infectious complications [1]. This disease is common in tropical regions and primarily affects healthy children and young adults. It may also occur in immunocompromised adult patients in temperate regions with underlying diseases such as human immunodeficiency virus infection, hematopoietic malignancy, diabetes mellitus, organ transplantation, malnutrition, and chronic kidney disease [2]. The common causative organisms are Staphylococcus aureus, Streptococcus spp., Group A Streptococcus, and Pseudomonas spp [2]. The lower extremities and trunk are most commonly affected, whereas occurrence in the chest wall is rare. Although proper antibiotic therapy is essential, most patients require surgical or radiological-guided drainage of muscle abscess [3]. Here, we report a case of pyogenic pectoral myositis caused by Morganella morganii in an elderly patient with type 2 diabetes.

## Case presentation

**Figure 1:**
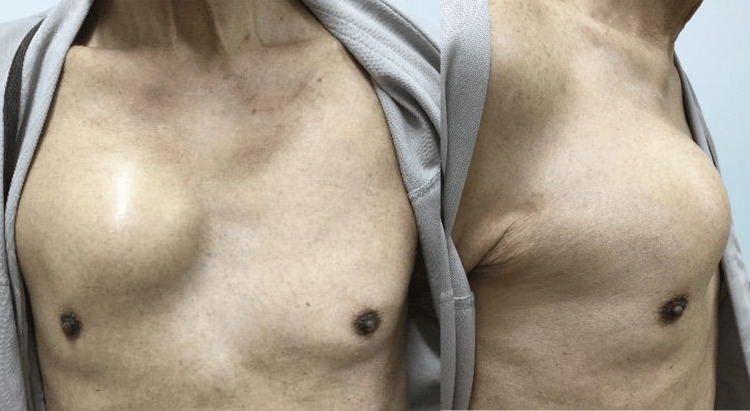
Chest findings at the initial examination. There was swelling in the right anterior thoracic region with a diameter of 10 cm.

The patient was an 82-year-old Japanese man with type 2 diabetes mellitus who had undergone stenting for myocardial infarction. He had no allergies, but had a history of past smoking. Four months prior to his visit to our hospital, the patient had become aware of pain in the right chest area, which gradually became swollen. One month before the visit, the pain and swelling had become more severe. At the hospital visit, there was swelling in the right anterior thoracic region with a diameter of 10 cm and pain in the same region. The same region was hard and the pulses were not palpable. No inflammatory findings such as redness were observed in the surrounding skin (Figure 1) and there was no history of chest trauma.

**Figure 2:**
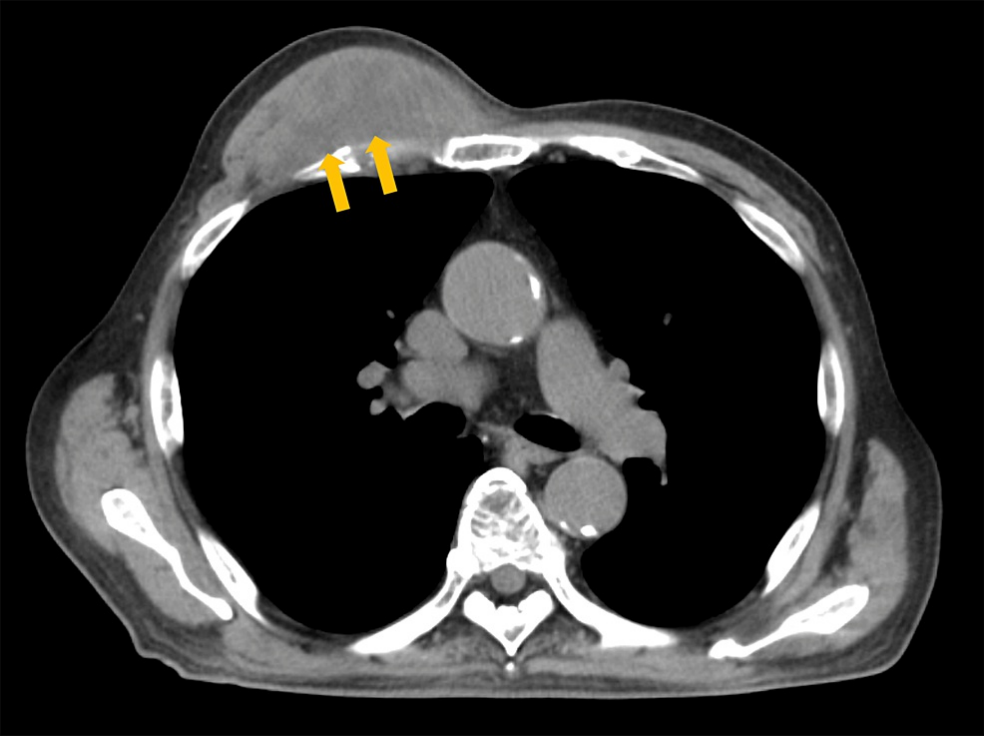
Chest CT findings. CT showed pectoral muscle swelling and a mosaic of low-absorption areas under the pectoralis major muscle (yellow arrow).

**Figure 3:**
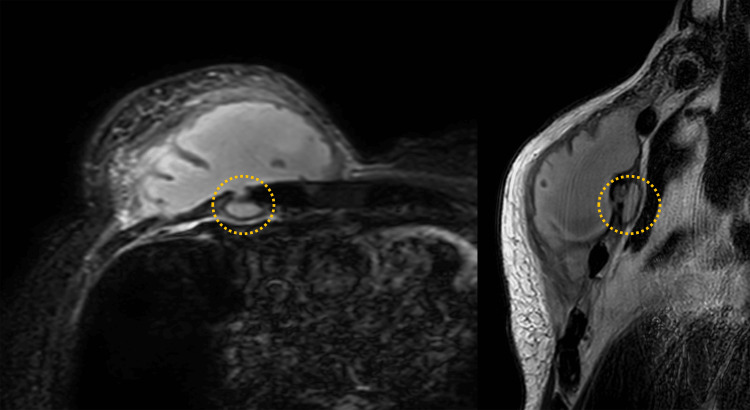
Chest MRI findings. Part of the lesion penetrated the chest wall and formed an abscess (yellow dotted circle) with surrounding pleural thickening.

On physical examination, his blood pressure was 133/64 mmHg, heart rate was 83 beats/min, and body temperature was 36.9℃. Blood tests showed a white blood cell count of 18,800/µL (reference range: 4,000-9,600/µL), C-reactive protein 9.34 mg/dL (reference range: <0.1 mg/dL) and an elevated inflammatory response. HbA1c was high, at 8.3% (reference range: 4.6-6.2%). A T-SPOT.TB test was negative. Initially, a sarcoma or other neoplastic lesion was suspected and a needle biopsy was performed. Pus was drained from the puncture site to collect wound culture. Needle biopsy results showed suppurative granulomatous inflammation and did not reveal any fungi or acid-fast bacteria. Computed tomography showed pectoral muscle swelling and a mosaic of low-absorption areas under the pectoralis major muscle (Figure 2). On magnetic resonance imaging (MRI), the lesion under the pectoralis major muscle gave a low signal on a T1-weighted image and a high signal on a T2-weighted image and STIR, suggesting abscess formation. In addition, part of the lesion had penetrated the chest wall and formed an abscess with surrounding pleural thickening (Figure 3).

These findings led to the diagnosis of pectoralis major pyomyositis and the patient was started on piperacillin/tazobactam (13.5 g/day). Although it was necessary to differentiate it from necrotizing fasciitis, it was considered negative in this case because of the slow progression of the disease, stable vital signs, and lack of skin findings such as blisters, bullae, and skin crepitus that are diagnostic features of necrotizing fasciitis [4].

**Figure 4:**
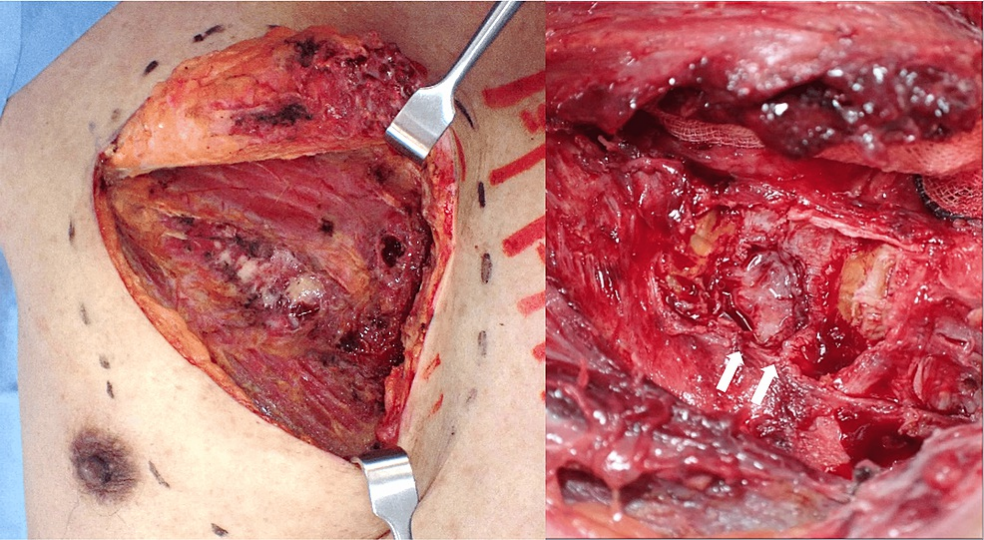
Surgical findings. The necrotic pectoralis major muscle was excised and the rotund cartilage was removed (white arrow).

Debridement was considered necessary due to a concern that the infection might progress to an intrathoracic abscess. In surgery, the necrotic pectoralis major muscle was excised, the abscess cavity was opened, and debridement was performed. The third rib cartilage was partially fused and the rotund cartilage was removed (Figure 4). After thorough wound irrigation, a suction drain was inserted under the pectoralis major muscle and the wound was closed.

Wound cultures at admission and surgery revealed Morganella morganii. After susceptibility testing (Table 1), the antibiotic was changed to cefmetazole sodium (2 g/day) on postoperative day (POD) 4, and to sulfamethoxazole (SMX)/trimethoprim (TMP) (800 mg/day SMX + 160 mg/day TMP) on POD 14.​​

**Table 1:** Drug susceptibility result MIC: Minimum inhibitory concentration, R: Resistant, S: Susceptible

Antimicrobial agent	MIC (㎍/mL)	Susceptibility
Ampicillin	≥ 32	R
Cefazolin	≥ 32	R
Cefpodoxime Proxetil	≤ 1	S
Ceftazidime	≤ 2	S
Imipenem/Cilastatin	=1	S
Amikacin	≤ 8	S
Gentamicin	≤ 2	S
Sulbactam/Ampicillin	= 8	S
Tazobactam/Piperacillin	≤ 4	S
Cefuroxime	≥ 32	R
Cefmetazole	≤ 8	S
Cefotaxime	≤ 1	S
Cefepime	≤ 1	S
Latamoxef	≤ 4	S
Aztreonam	≤ 2	S
Meropenem	= 0.25	S
Levofloxacin	≤ 1	S
Ciprofloxacin	≤ 0.5	S
Sulfamethoxazole-Trimethoprim	≤ 19	S

**Figure 5:**
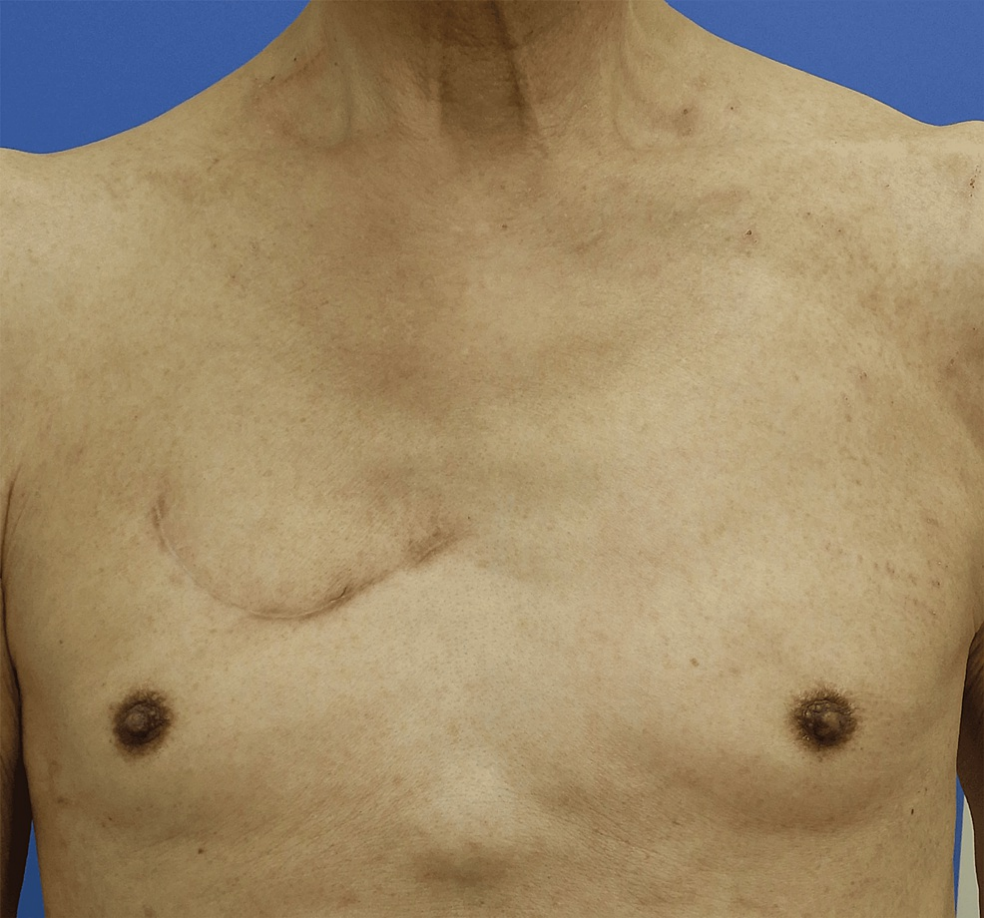
Chest findings at seven months postoperatively. There has been no recurrence of the abscess for eight months postoperatively.

The postoperative course was good and the patient was discharged on POD 16. In this case, the abscess extended into the rib cartilage, and long-term antibiotic therapy was considered necessary, so SMX/TMP oral medication was continued for one month. There has been no recurrence of the abscess in eight months postoperatively (Figure 5).

## Discussion

Pyogenic myositis is common in tropical regions in children and young adults without underlying disease. In recent years, this disease has also been reported in temperate regions, often in elderly people with underlying diseases such as human immunodeficiency virus infection, hematopoietic tumors, diabetes mellitus, organ transplantation, malnutrition, and chronic kidney disease [2]. Methicillin‐sensitive Staphylococcus aureus (MSSA) (46%), methicillin‐resistant Staphylococcus (MRSA) (20%), Streptococcal spp. (11%), Group A streptococcus (4.8%), and Pseudomonas spp. (3.6%) are the common causative organisms [2]. The common sites of infection are the quadriceps, psoas, and iliopsoas muscles [3]. Shepherd has proposed several theories for the pathogenesis of pyomyositis, including trauma, parasites, viruses, and nutritional disorders, but there is still no established etiology [5].

The case presented here is an example of pectoralis major pyomyositis that arose in a temperate climate in an elderly patient with diabetes. A literature search of PubMed using the search terms “pectoralis pyomyositis” indicated that nine English cases have been reported since 2000, including our case (Table 2) [6-13].

**Table 2:** Pectoralis pyomyositis cases MSSA: Methicillin‐sensitive Staphylococcus aureus, MRSA: Methicillin‐resistant Staphylococcus

Case	Ref.	Author	Year	Age / Sex	Predisposing factors	Bacterium	Abscess formation	Surgery, Drainage
1	[6]	López-Rodríguez et al.	2008	44 / male	physical effort	MSSA	+	-
2	[7]	Wu et al.	2012	88 / male	tooth extraction	MSSA	+	Surgical debridement
3	[8]	Saad et al.	2018	54 / female	-	MSSA	-	-
4	[9]	Tatsuno et al.	2020	63 / male	end-stage renal disease	MRSA	+	CT-guided drainage, Surgical debridement
5	[10]	Rayzah	2020	60 / male	diabetes mellitus	MRSA	+	Surgical debridement
6	[11]	Seyfi et al.	2021	43 / male	intravenous drug use	not detected	+	Ultrasound-guided drainage
7	[12]	Dalal et al.	2021	77 / male	diabetes mellitus	Bacteroides fragilis	+	Surgical debridement
8	[13]	Kollia et al.	2023	45 / male	intravenous drug use	MRSA	+	incisional drainage
9		Our case	2024	82 / male	diabetes mellitus	Morganella morganii	+	Surgical debridement

Patients included eight males and one female with a mean age of 61.8 years. Three had diabetes mellitus in their medical history and one had renal dysfunction. Two were intravenous drug abuser. Physical effort and tooth extraction were considered triggers for one patient each. Eight of the nine cases showed abscess formation, and drainage or debridement was performed in seven of them. The pathogen was MSSA in three cases, MRSA in three, Bacteroides fragilis in one, Morganella morganii in one, and an unknown type in one. Pyogenic myositis of the pectoralis major muscle is rare, and there is only one report of pyogenic myositis caused by Morganella morganii in the right anterior tibialis muscle, which occurred in a patient with acquired immunodeficiency syndrome [14]. Morganella morganii is a Gram-negative rod belonging to the Enterobacteriaceae family and is a multidrug-resistant bacterium that is naturally resistant to penicillin and first- and second-generation cephem antibiotics [15]. However, it is not particularly virulent, and in our case, a weakened immune system due to diabetes may have contributed to the onset of the disease.

Pyomyositis progresses from stage I (muscle spasms, progressive pain, low-grade fever) to stage II (abscess formation) to stage III (sepsis) [3]. The mortality rate is 0.89-14.0%, so early detection and treatment are important [6]. Pyomyositis may exhibit symptoms similar to necrotizing fasciitis, making the diagnosis difficult. Although swelling, erythema, and disproportionately severe pain can also be present in necrotizing fasciitis, blisters, bullae, and skin crepitus are diagnostic features of necrotizing fasciitis [4]. MRI is helpful for differentiating between pyomyositis and necrotizing fasciitis. A diffuse hyperintense signal in muscles on fat-suppressed T2-weighted images, diffuse contrast enhancement of muscle, thick irregular enhancement of the deep fascia, and intramuscular abscess are MRI findings characteristic of pyomyositis [16]. Antimicrobial therapy is effective, but drainage may be necessary if abscess formation is observed. A focus should be placed on antimicrobials targeting MSSA, but empirical antimicrobial therapy covering MRSA and Gram-negative and anaerobic bacteria should also be considered. Cases have been reported in which chest wall pyomyositis developed into pleural empyema [17, 18] or mediastinitis [19]. Surgical drainage should be performed promptly in cases with abscess formation, since extension to deeper organs leads to more severe symptoms and may be refractory to treatment.

## Conclusions

We experienced a case of pectoralis major pyomyositis in an elderly Japanese patient with type 2 diabetes. Pyogenic myositis has been increasingly reported in temperate regions, especially in elderly patients with underlying diseases. Since treatment with antimicrobial agents alone can be difficult, and early diagnosis and treatment can determine prognosis, surgical drainage should be performed promptly when abscess formation is observed.
